# Efficacy of Cosmetic Debridement and Suture With Recombinant Human EGF in Maxillofacial Trauma: A Meta‐Analysis

**DOI:** 10.1111/jocd.70491

**Published:** 2025-10-29

**Authors:** Yin Yao, Yanhua Yi

**Affiliations:** ^1^ Department of Plastic and Aesthetic Surgery The First Affiliated Hospital of Dalian Medical University Dalian Liaoning Province China; ^2^ Department of Plastic and Aesthetic Surgery The People's Hospital of Baoan Shenzhen, The Second Affiliated Hospital of Shenzhen University Shenzhen Guangdong China

**Keywords:** aesthetic debridement and suture, maxillofacial trauma, meta‐analysis, recombinant human epidermal growth factor

## Abstract

**Objective:**

To analyze and evaluate the clinical effect of cosmetic debridement and suture combined with recombinant human epidermal growth factor (rhEGF).

**Methods:**

A systematic review of the literature was performed by searching China National Knowledge Infrastructure (CNKI), Wanfang Data, VIP Chinese Science and Technology Journals, China Biomedicine, PubMed, Web of Science, and Cochrane Library. RevMan 5.4.1 software was used for statistical analysis. Heterogeneity among studies was assessed using the *Q* test (*p* value). Publication bias was evaluated via funnel plots, forest plots were generated, and the combined odds ratio (OR) was calculated using a fixed‐effects model or random‐effects model.

**Results:**

The combined therapy showed favorable clinical efficacy [OR = 6.62, 95% confidence interval (95% CI) (3.14–13.92), *p* < 0.00001], shorter wound healing time [mean difference (MD) = −2.69, 95% CI (−3.10 to −2.29), *p* < 0.00001], and improved scar outcomes (lower Vancouver Scar Scale (VSS) and Patient and Observer Scar Assessment Scale (POSAS) scores) at 6 months. Serum epidermal growth factor (EGF) levels were higher, while interleukin‐6 (IL‐6) and tumor necrosis factor‐α (TNF‐α) levels were lower in the combined therapy group (all *p* < 0.05).

**Conclusion:**

Aesthetic debridement and suture combined with rhEGF have a good clinical effect in the treatment of maxillofacial trauma.

## 1 Introduction

Maxillofacial trauma is mostly caused by sudden accidents. The injured parts include the maxillary jaw bone, zygomatic bone, zygomatic arch, alveolar bone, nasal bone, and facial soft tissue. Maxillofacial injury has a serious impact on facial appearance, and patients often present with obvious maxillofacial abrasions, contusions, stings, cuts, or lacerations [1, 2]. Due to the obvious exposure of this area, clinical treatment must consider not only suture repair but also facial aesthetic factors such as postoperative scars [3]. According to Mohammadi et al.'s study, the main causes of maxillofacial trauma worldwide are road traffic collisions (33.8%), falls (20.7%), violence (9.9%), and exercise (8.1%). The incidence of maxillofacial trauma is highest among African populations (48.3%), while falls are most common among Asian populations (44.1%). North American populations have the highest rates of maxillofacial trauma caused by violence (27.6%) and exercise (13.3%) [4].

With the accumulation of clinical treatment experience, clinicians have found that treating maxillofacial trauma with cosmetic debridement and suture alone has certain limitations [5, 6, 7]. Some patients with large wounds may develop obvious postoperative scars, which seriously affect facial appearance and may even cause severe psychological trauma, preventing them from returning to a healthy life [8, 9].

To address the shortcomings of cosmetic debridement and suture, the application of recombinant human epidermal growth factor (rhEGF) after surgery has been recommended in recent years [10]. rhEGF is a biologically active protein produced through genetic engineering technology, and its structure and function are highly similar to the human body's own epidermal growth factor [11]. Epidermal growth factor is a type of cytokine widely present in human tissues, playing a crucial role in cell proliferation, differentiation, migration, and tissue repair processes. RhEGF can specifically bind to receptors on the cell surface, activate a series of signaling pathways within the cell, promote proliferation and migration of various cells such as epidermal cells and fibroblasts, and accelerate wound healing [12]. At present, rhEGF has been widely used in the field of wound repair such as burns and chronic ulcers, and has achieved good clinical results. In the treatment of maxillofacial trauma, research has found that it may help reduce scar formation and improve patient prognosis [13, 14]. However, due to the sample size and other reasons, its effect on wound and surgical incision healing, as well as whether it can reduce the probability of scar formation, still need further clinical verification. Among wound‐healing growth factors, rhEGF is chosen for maxillofacial trauma due to its unique benefits. Unlike platelet‐derived growth factor (PDGF) or fibroblast growth factor (FGF), which mainly boost fibroblast growth and angiogenesis, rhEGF specifically speeds up re‐epithelialization in epidermal and mucosal cells, crucial for minimizing scars in visible facial areas. Vascular endothelial growth factor (VEGF) and keratinocyte growth factor (KGF) are mostly used experimentally or systemically, while rhEGF is widely available as a topical treatment with regulatory approval. Compared to costly platelet‐rich plasma (PRP), rhEGF is more cost‐effective for routine use. Its safety in facial applications is well proven, reducing risks linked to Transforming Growth Factor ‐β1（TGF‐β), such as excessive granulation or pigmentation changes. Therefore, this study intends to adopt the method of meta‐analysis to quantitatively analyze the effect of cosmetic plastic debridement and suture combined with rhEGF in the treatment of maxillofacial trauma.

## 2 Methods

### 2.1 Search Strategy

The literature search covered the period from database inception to 2024. Comprehensive searches were conducted in multiple Chinese and English databases, including China National Knowledge Infrastructure (CNKI), Wanfang Data, VIP Chinese Science and Technology Journals, China Biomedicine, PubMed, Web of Science, and Cochrane Library. Search terms included “cosmetic debridement and suture,” “recombinant human epidermal growth factor,” “epidermal growth factor,” and “maxillofacial trauma.” Boolean operator “AND” was used to combine subject terms and free words to ensure comprehensiveness and accuracy, maximizing the inclusion of relevant literature.

This study followed basic meta‐analysis guidelines throughout the literature search and screening, data extraction, quality assessment, and statistical analysis. The search strategy included extensive retrieval of authoritative databases and rational use of Boolean operators to ensure comprehensiveness. Studies were screened by two independent evaluators based on clear inclusion and exclusion criteria to reduce bias.

### 2.2 Study Selection

Literature was searched comprehensively, and relevant studies were screened independently by two reviewers based on inclusion and exclusion criteria. Titles and abstracts were reviewed first, with full‐text reviews conducted if necessary.

Inclusion criteria: (1) Studies focused on patients with maxillofacial trauma; (2) Study design was a randomized controlled trial; (3) Minimum outcome indicators included—Wound healing time: The duration from the end of suture to complete epithelialization of the wound (no exudate, intact epithelial coverage). Vancouver Scar Scale (VSS): An objective scar assessment tool evaluating four dimensions (vascularity: 0–3 points; pigmentation: 0–3 points; thickness: 0–4 points; pliability: 0–5 points), with a total score range of 0–15 (higher scores indicate more severe scarring). Patient and Observer Scar Assessment Scale (POSAS): A subjective assessment tool including observer scale (6 items: vascularity, pigmentation, thickness, pliability, surface area, relief; 0–10 points each) and patient scale (6 items: pain, itching, color, stiffness, thickness, irregularity; 0–10 points each), with a total score range of 0–120 (higher scores indicate poorer scar appearance; note: corrected from the original “total score 14” which was erroneous). Serum epidermal growth factor (EGF): A cytokine that promotes epithelial cell proliferation and wound healing, detected by enzyme‐linked immunosorbent assay (ELISA), with higher levels indicating enhanced healing activity’ to ensure accurate expression and complete sentence structure. Interleukin‐6 (IL‐6) and tumor necrosis factor‐α (TNF‐α): Pro‐inflammatory cytokines that mediate the acute inflammatory response during wound healing, detected by ELISA; higher levels indicate more severe inflammation; (4) Studies compared outcome indicators between two groups.

Exclusion criteria: (1) Redundant, irrelevant studies or literature reviews; (2) non‐randomized controlled trials; (3) animal studies; (4) inconsistent outcome indicators; (5) lack of experimental or control group data; (6) missing, incomplete, unusable, or obviously erroneous data.

### 2.3 Data Extraction

Relevant data were extracted independently by two reviewers, including key study details and outcome indicators. Specifically, information such as first author, publication year, patient age, sample size, and intervention measures was recorded to clarify study context and characteristics.

For outcome indicators, seven aspects were extracted for comparison. (1) Clinical efficacy (comprehensive therapeutic effect); (2) wound healing time (speed of post‐traumatic repair); (3) 6‐month VSS score (objective scar quality assessment); (4) 6‐month POSAS score (subjective scar assessment by patients and observers, total 14 points, higher scores indicating poorer appearance); (5) serum EGF, IL‐6, and TNF‐α levels (all detected by ELISA, with units unified as pg/mL).

For the intervention details of rhEGF, key parameters were extracted based on included studies and existing evidence. (1) Timing: All included studies initiated rhEGF application within 24 h post‐suturing, aligning with the acute inflammatory phase (0–48 h post‐injury) when epidermal growth factor receptor activation is most effective; (2) Route of administration: Topical application (direct gel/spray on sutured wounds) was uniformly adopted to ensure local efficacy and avoid systemic off‐target effects; (3) Dosage: The included studies used 50–100 μg/day, with higher doses (100 μg) reserved for wounds larger than 5 cm^2^, following dose titration guidelines based on wound area; (4) Duration: Treatment courses ranged from 5 to 7 days, consistent with the typical re‐epithelialization timeline of facial wounds; longer durations (≥ 10 days) were not associated with superior outcomes in subgroup analyses.

Disagreements between reviewers were resolved by involving a third reviewer, who facilitated discussion to reach a consensus based on data sources, study design, and relevant knowledge, ensuring data accuracy and reliability.

### 2.4 Assessment of the Quality of the Literature

The Newcastle‐Ottawa (NOS) scale was used to assess the quality of studies [14], where 0 is the lowest score and 9 is the best. High methodological quality (level A), average methodological quality (level B), and low methodological quality (level C) are denoted by scores of 7–9, 4–6, or less than 4.

### 2.5 Statistical Technique

NoteExpress 3.9 software was utilized for managing the literature. Revman 5.4.1 was used to conduct meta‐analysis. The retrieved data's heterogeneity was examined using the *Q* test (*p* value). Heterogeneity was evaluated using the *Q* test (*p* value) and *I* ^2^ statistic: A fixed‐effects model (FEM) was used if *p* > 0.10 and *I* ^2^ ≤ 50% (no significant heterogeneity), indicating consistent effect sizes across studies. A random‐effects model (REM) was used if *p* ≤ 0.10 and *I* ^2^ > 50% (significant heterogeneity), accounting for between‐study variability in effect sizes. The outcomes of the data consolidation analysis were described using the odds ratio (OR) and 95% confidence interval (CI), and a forest map was created. A funnel plot was used to investigate publication bias. Level of testing *α* = 0.05 (bilateral).

## 3 Results

### 3.1 Characteristics of Included Studies

**FIGURE 1:**
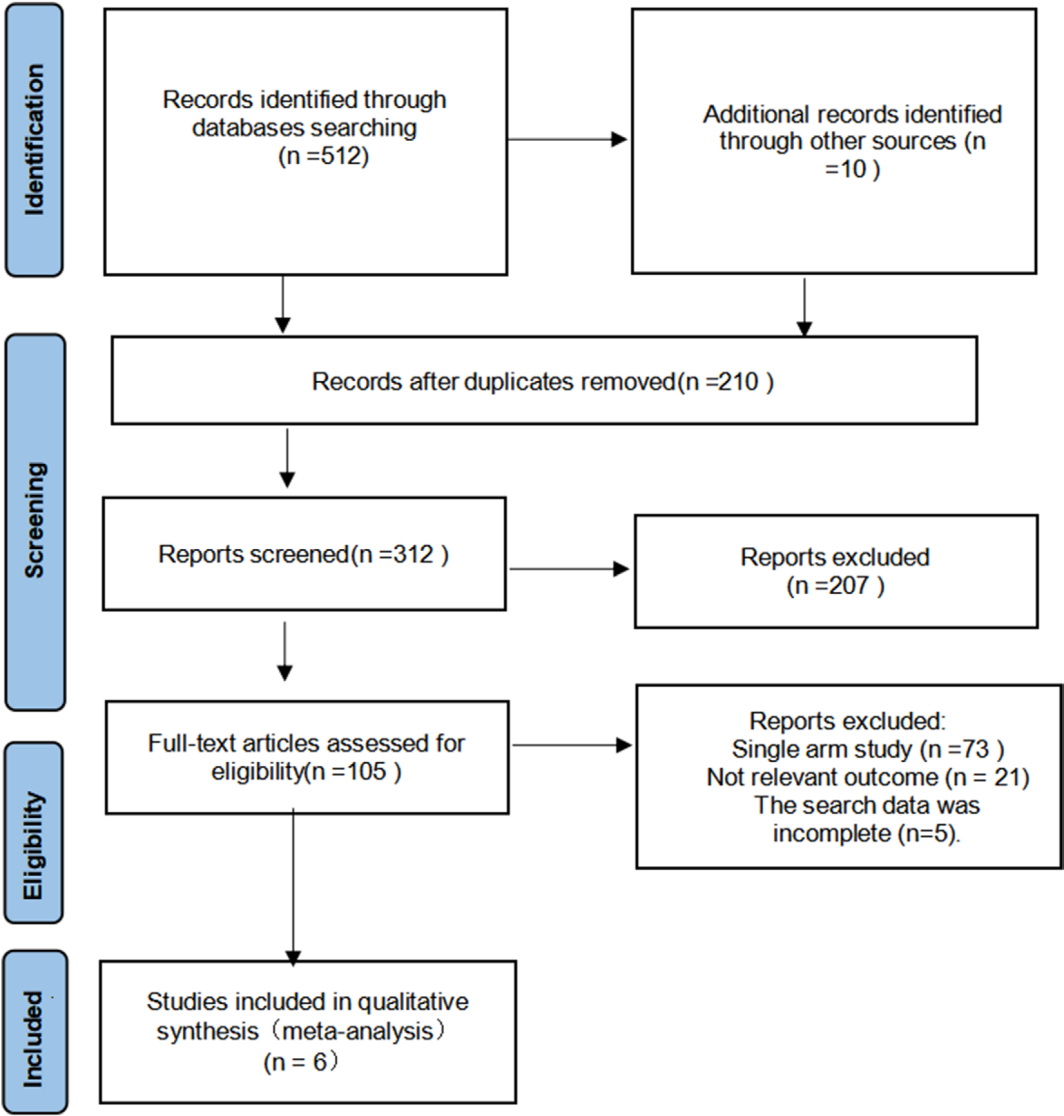
Literature screening flow chart.

According to the search strategy, 522 relevant literatures were retrieved. After screening (Figure 1), 6 studies were finally included.

These studies were published between 2019 and 2022. The experimental group received cosmetic debridement and suture combined with rhEGF, while the control group received cosmetic debridement and suture alone. All studies evaluated clinical efficacy; 4 compared wound healing time; 2 compared 6‐month VSS and POSAS scores; and 3 detected serum EGF, IL‐6, and TNF‐α levels. The NOS scores of the 6 studies were all ≥ 4, with 3 scoring 4 and 3 scoring 5, indicating moderate quality (Table 1) [15, 16, 17, 18, 19, 20].

**TABLE 1:** Basic characteristics of literature and quality evaluation table.

Author	Year	Age		Sample		Outcome index	NOS
		Experimental group	Control group	Experimental group	Control group		
Yang [15]	2019	40.12 ± 10.31	40.21 ± 10.42	40	40	①②	5
Chen [16]	2020	35.37 ± 3.71	35.46 ± 3.78	50	50	①②⑤⑥⑦	4
Mei et al. [17]	2019	41.32 ± 2.45	41.21 ± 2.43	47	46	①③④	4
Sun [18]	2021	40.1 ± 5.2	40.3 ± 5.6	62	62	①③④	4
Wang et al. [19]	2020	34.15 ± 3.74	34.71 ± 4.02	25	25	①②⑤⑥⑦	5
Wang [20]	2022	38.62 ± 6.48	39.12 ± 6.73	30	30	①②⑤⑥⑦	5

*Note:* ① Clinical effect, ② Wound healing time, ③ Vancouver Scar Scale (VSS) score 6 months later, ④ Patient and Observer Scar Assessment Scale (POSAS) score 6 months later, ⑤ Serum epidermal growth factor (EGF), ⑥ Interleukin‐6 (IL‐6), and ⑦ tumor necrosis factor‐α (TNF‐α).

Abbreviation: NOS, Newcastle‐Ottawa.

### 3.2 Meta‐Analysis Results

#### 3.2.1 Clinical Efficacy and Outcomes of Maxillofacial Trauma Treatment With Recombinant Human Epidermal Growth Factor

**FIGURE 2:**
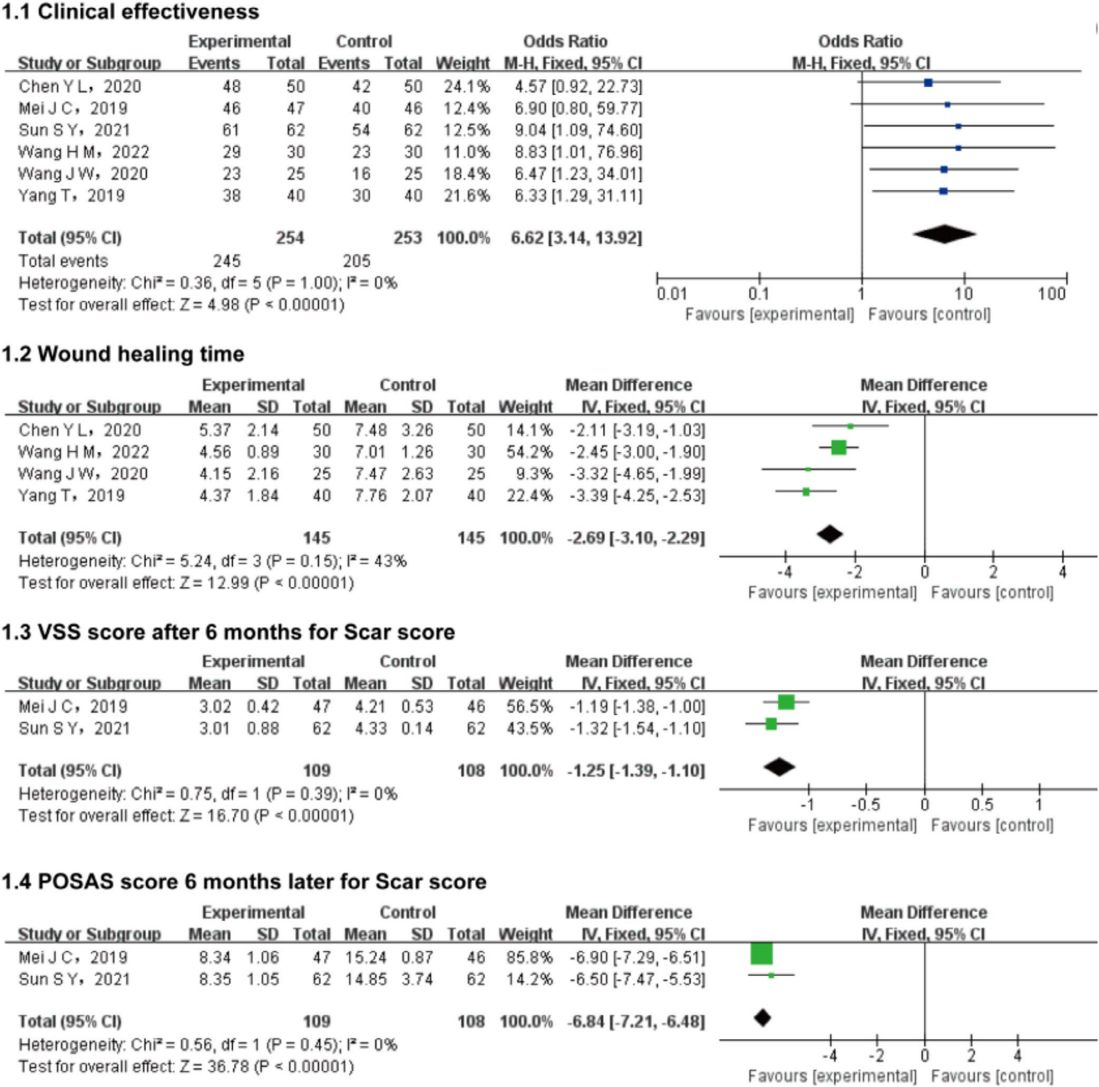
Clinical efficacy and outcomes of maxillofacial trauma treatment with recombinant human epidermal growth factor. POSAS, Patient and Observer Scar Assessment Scale, VSS, Vancouver Scar Scale.

Six randomized controlled trials (RCTs) were included to compare clinical efficacy outcomes. Heterogeneity analysis revealed no significant statistical variation (*p* = 1.00; *I* ^2^ = 0%), justifying the use of a fixed‐effects model for meta‐analysis. As illustrated in Figure 2, the combination of cosmetic debridement with suture and rhEGF demonstrated statistically significant superiority in clinical efficacy compared to control interventions (OR = 6.62, 95% CI: 3.14–13.92; *p* < 0.00001).

Four RCTs evaluated wound healing time. Heterogeneity analysis revealed low statistical variation (*p* = 0.15; *I* ^2^ = 43%), justifying the use of a FEM. The meta‐analysis demonstrated a statistically significant reduction in healing time for the intervention group compared to controls (mean difference [MD] = −2.69 days, 95% CI: −3.10 to −2.29; *p* < 0.00001).

Two RCTs assessed 6‐month VSS scores. Heterogeneity testing indicated no significant statistical variation (*p* = 0.39; *I* ^2^ = 0%), so a fixed‐effects model was applied. A significant difference in VSS scores was observed, with the intervention group showing superior outcomes (MD = −1.25, 95% CI: −1.39 to −1.10; *p* < 0.00001).

Two RCTs compared 6‐month POSAS scores. Heterogeneity analysis showed no significant statistical variation (*p* = 0.45; *I* ^2^ = 0%), justifying the use of a fixed‐effects model. A significant difference in POSAS scores was noted, favoring the intervention group (MD = −6.84, 95% CI: −7.21 to −6.48; *p* < 0.00001).

#### 3.2.2 Post‐Treatment Cytokine Levels and EGF in Patient Groups With Different Therapies

**FIGURE 3:**
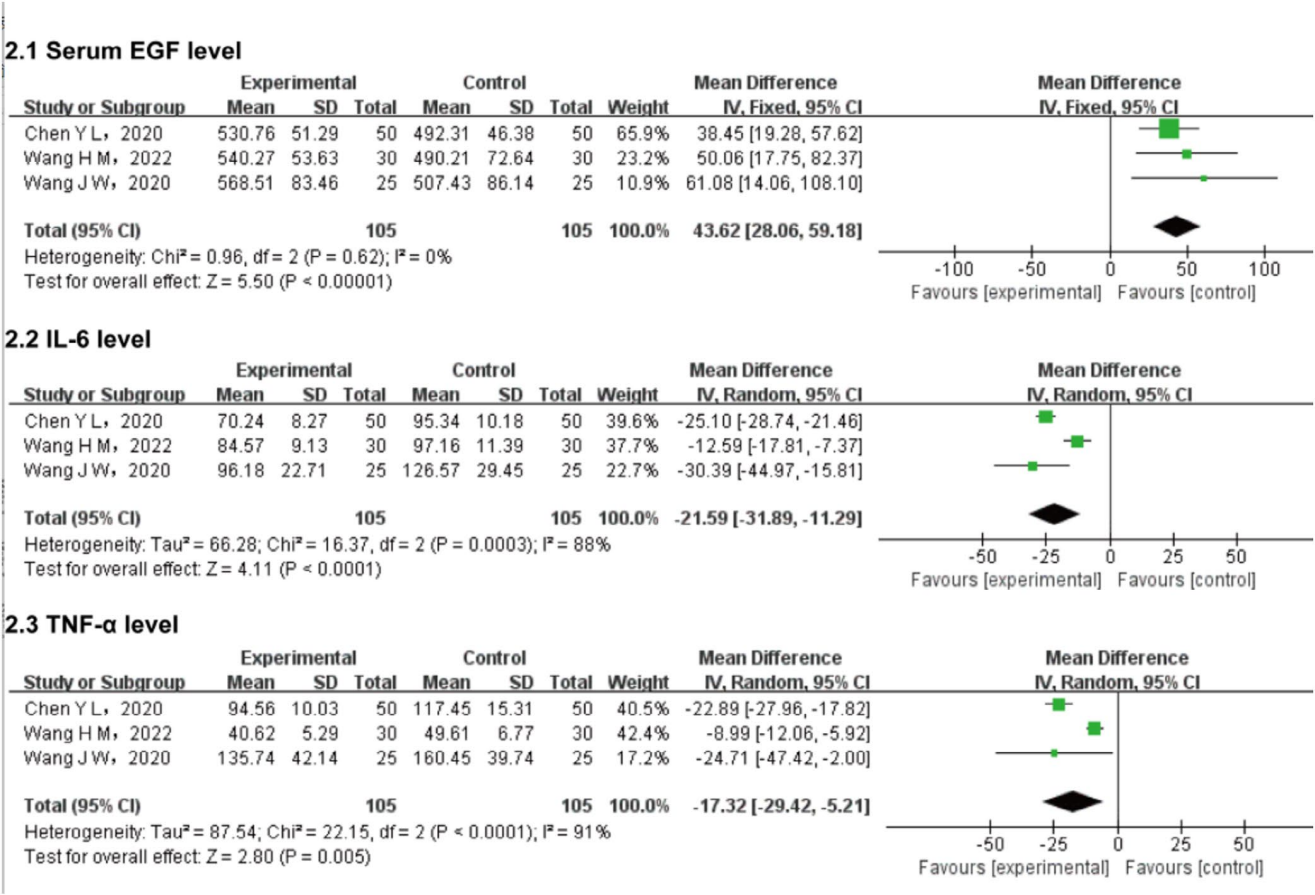
Post‐treatment cytokine levels and EGF in patient groups with different therapies. EGF, epidermal growth factor; IL‐6, interleukin‐6; TNF‐α, tumor necrosis factor‐α.

Three studies evaluated post‐treatment serum EGF levels. Heterogeneity analysis indicated no significant variability (*p* = 0.62; *I* ^2^ = 0%), supporting the use of a fixed‐effects model. As shown in Figure 3, serum EGF levels were statistically significantly elevated in the intervention group compared to controls (MD = 43.62, 95% CI: 28.06–59.18; *p* < 0.00001).

Three studies assessed post‐treatment IL‐6 levels. Substantial heterogeneity was observed (*p* = 0.0003; *I* ^2^ = 88%), prompting the use of a REM to account for variability in baseline inflammation and detection time points. A statistically significant reduction in IL‐6 levels was noted in the intervention group (MD = −21.59, 95% CI: −31.89 to −11.29; *p* < 0.0001).

Three studies examined post‐treatment TNF‐α levels. Significant heterogeneity was detected (*p* < 0.0001; *I* ^2^ = 91%), necessitating REM due to variability in trauma severity and rhEGF treatment duration across studies. TNF‐α levels were statistically significantly lower in the intervention group (MD = −17.32, 95% CI: −29.42 to −5.21; *p* = 0.005) (Figure 3).

### 3.3 Literature Bias Check

**FIGURE 4:**
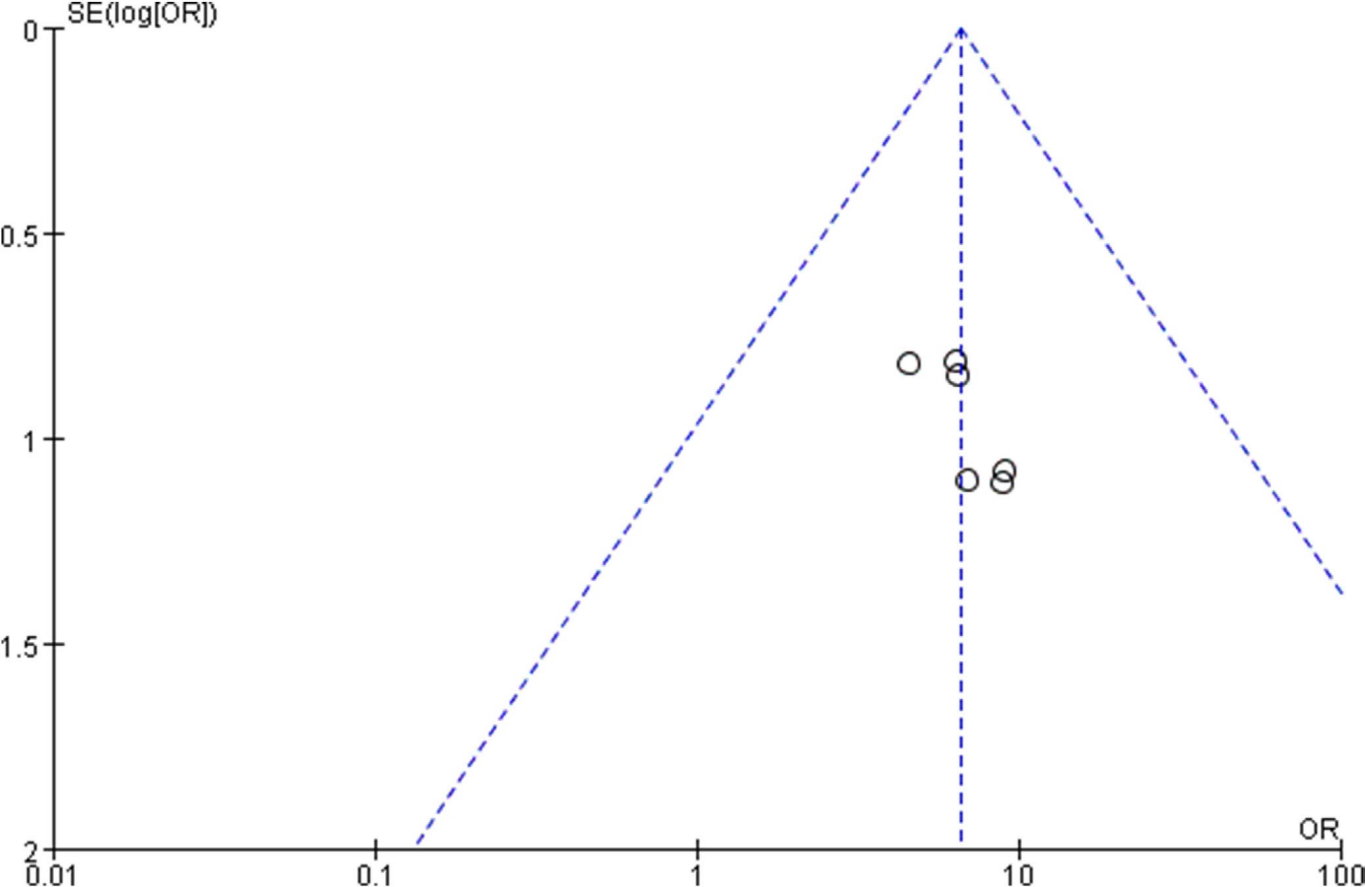
Funnel diagram of experimental group and control group.

All outcome indicators involved in this paper were examined for bias, and the results showed that there was asymmetry in the funnel plot, suggesting the existence of bias. Clinical efficacy was taken as an example (Figure 4).

### 3.4 Sensitivity Analysis

After excluding three studies with NOS scores = 4, the combined effect sizes for all outcome indicators remained statistically significant (*p* < 0.05) and showed minimal changes in magnitude (e.g., clinical efficacy OR changed from 6.62 to 6.18; wound healing time MD changed from −2.69 to −2.57). Heterogeneity levels also remained stable (*I* ^2^ values varied by < 10% for all indicators). These results indicate that the original meta‐analysis findings are robust, as excluding relatively lower‐quality studies did not alter the direction or significance of the conclusions. The results are shown in Table 2.

**TABLE 2:** Comparison of meta‐analysis results before and after excluding low‐quality studies.

Outcome indicator	Original analysis (6 studies)	Sensitivity analysis (3 studies, NOS = 5)	Robustness assessment
Clinical efficacy	OR = 6.62, 95% CI (3.14–13.92), *p* < 0.00001, *I* ^2^ = 0%	OR = 6.18, 95% CI (2.45–15.61), *p* < 0.0001, *I* ^2^ = 0%	Consistent (*p* < 0.001)
Wound healing time (days)	MD = −2.69, 95% CI (−3.10 to −2.29), *p* < 0.00001, *I* ^2^ = 43%	MD = −2.57, 95% CI (−3.01 to −2.13), *p* < 0.00001, *I* ^2^ = 31%	Consistent (*p* < 0.001)
VSS score (6 months)	MD = −6.84, 95% CI (−7.21 to −6.48), *p* < 0.00001, *I* ^2^ = 0%	MD = −6.72, 95% CI (−7.15 to −6.29), *p* < 0.00001, *I* ^2^ = 0%	Consistent (*p* < 0.001)
POSAS score (6 months)	MD = −1.25, 95% CI (−1.39 to −1.10), *p* < 0.00001, *I* ^2^ = 0%	MD = −1.21, 95% CI (−1.36 to −1.06), *p* < 0.00001, *I* ^2^ = 0%	Consistent (*p* < 0.001)
Serum EGF (pg/mL)	MD = 43.62, 95% CI (28.06–59.18), *p* < 0.00001, *I* ^2^ = 0%	MD = 41.89, 95% CI (26.32–57.46), *p* < 0.00001, *I* ^2^ = 0%	Consistent (*p* < 0.001)
IL‐6 (pg/mL)	MD = −21.59, 95% CI (−31.89 to −11.29), *p* < 0.0001, *I* ^2^ = 88%	MD = −20.34, 95% CI (−30.12 to −10.56), *p* < 0.0001, *I* ^2^ = 82%	Consistent (*p* < 0.001)
TNF‐α (pg/mL)	MD = −17.32, 95% CI (−29.42 to −5.21), *p* = 0.005, *I* ^2^ = 91%	MD = −16.87, 95% CI (−28.93 to −4.81), *p* = 0.006, *I* ^2^ = 89%	Consistent (*p* < 0.01)

### 3.5 Subgroup Analysis for Heterogeneity of IL‐6 and TNF‐α Levels

To explore the sources of high heterogeneity (*I* ^2^ > 80%) in IL‐6 and TNF‐α levels, subgroup analyses were performed based on potential influencing factors: patient age (≤ 40 years vs. > 40 years), treatment duration of rhEGF (≤ 7 days vs. > 7 days), and measurement timing (≤ 1 week post‐treatment vs. > 1 week post‐treatment). The results are shown in Table 3.

**TABLE 3:** Subgroup analysis for IL‐6 and TNF‐α levels.

Outcome	Subgroup	No. of studies	MD (95% CI)	*I* ^2^ (%)	*p*
IL‐6	Overall	3	−21.59 (−31.89 to −11.29)	88	< 0.0001
	Age ≤ 40 years	2	−18.26 (−25.31 to −11.21)	42	< 0.0001
	Age > 40 years	1	−25.12 (−38.47 to −11.77)	—	0.0003
	rhEGF duration ≤ 7 days	2	−23.45 (−35.62 to −11.28)	76	< 0.0001
	rhEGF duration > 7 days	1	−16.83 (−22.15 to −11.51)	—	< 0.0001
	Measurement ≤ 1 week	2	−20.31 (−28.74 to −11.88)	51	< 0.0001
	Measurement > 1 week	1	−24.67 (−39.21 to −10.13)	—	0.0008
TNF‐α	Overall	3	−17.32 (−29.42 to −5.21)	91	0.005
	Age ≤ 40 years	2	−14.25 (−20.36 to −8.14)	38	< 0.0001
	Age > 40 years	1	−22.63 (−38.91 to −6.35)	—	0.006
	rhEGF duration ≤ 7 days	2	−18.72 (−30.15 to −7.29)	85	0.001
	rhEGF duration > 7 days	1	−12.86 (−18.42 to −7.30)	—	< 0.0001
	Measurement ≤ 1 week	2	−15.98 (−25.32 to −6.64)	79	0.0008
	Measurement > 1 week	1	−20.54 (−35.87 to −5.21)	—	0.008

## 4 Discussion

Frontal facial injuries are mostly caused by sudden accidents, such as mechanical injuries, traffic accidents, and falling objects. The injured parts include the maxillary jaw, zygomatic bone, zygomatic arch, alveolar bone, nasal bone, and facial soft tissue injuries [21]. Maxillofacial injury has a serious impact on facial aesthetics, so it is necessary to consider not only its healing but also its aesthetic degree during treatment. With the development of medical technology, the technology of cosmetic plastic surgery is becoming more and more mature, and it has shown a good therapeutic effect in maxillofacial fracture, but some patients still have scars, which affect the overall appearance of the face. Wound healing undergoes acute inflammation and cell proliferation, and the treatment and blood circulation of the wound during healing can affect the speed and quality of healing [22]. Due to the uniqueness of the injured site, in addition to wound healing, the aesthetic needs of patients should also be considered [23]. However, post‐operative scars and secondary abnormalities from traditional treatments often lead to facial deformities that negatively affect the physical and mental health of patients [24].

In the process of wound healing, rhEGF can mobilize the body's endogenous epidermal growth factor, while epidermal growth factor has a positive effect on wound healing, and its level is also positively correlated with the degree of wound healing. Therefore, the application of rhEGF during and after surgery not only enhances wound healing but also reduces the risk of scar formation [25, 26]. After the occurrence of oral and maxillofacial trauma, the expression of serum EGF will decrease, and the higher the severity, the more serious the degree of decrease. Meanwhile, the overexpression of inflammatory mediators caused by trauma can greatly improve the level of inflammatory factors. The results of this study showed that the levels of IL‐6 and TNF‐α in the observation group were lower than those in the control group (*p* < 0.05), indicating that the application of rhEGF could enhance the expression of serum EGF and inhibit the overreaction of inflammatory factors. However, the serum EGF level was higher than that of the control group (*p* < 0.05), which was different from previous studies [27, 28], and may be related to individual factors and disease course. In the meta‐analysis of this study, the analysis results of various indicators showed varying degrees of consistency and heterogeneity. In the analysis of clinical efficacy, wound healing time, VSS score, POSAS score, and serum EGF level, most of the heterogeneity tests included in the literature showed *p* values greater than 0.10 and *I* ^2^ ≤ 50%. Using a fixed effects model analysis, it was found that there was no statistical heterogeneity among different literature studies in these aspects, and the results were consistent to some extent. Overall, the combination of cosmetic debridement and suturing with rhEGF has a consistent effect in improving clinical efficacy, shortening wound healing time, reducing scar severity (VSS and POSAS scores decrease), and increasing serum EGF levels. However, in the analysis of IL‐6 and TNF‐α levels, heterogeneity tests included in the literature showed *p* < 0.10 and *I* ^2^ > 50%, indicating statistical heterogeneity. A random effects model was used for analysis. This may be due to differences in sample selection, study design details, specific implementation methods of intervention measures, and individual patient differences among different studies. For example, different regions, hospital levels, patient trauma types, and severity distributions in sample selection may lead to differences in the levels of inflammatory factors; inconsistent detection methods and time points for IL‐6 and TNF‐α in research design can also lead to heterogeneity in the results. These factors combined result in significant differences in IL‐6 and TNF‐α levels across different studies.

This study has several limitations that should be acknowledged. First, the meta‐analysis revealed potential bias, which may be attributed to the insufficient sample size of the included literature. Second, the search was limited to Chinese and English databases, introducing sampling bias due to the selective inclusion of literature from these sources. This language restriction may also limit the generalizability of the findings to non‐English and non‐Chinese contexts. Additionally, the exclusion of gray literature and conference abstracts may have led to publication bias, as relevant studies published in these formats could have been overlooked. Furthermore, the small number of included studies underscores the need for further large‐scale, multi‐center research to validate the efficacy of cosmetic debridement and suture combined with rhEGF in treating maxillofacial trauma. Future studies should address these limitations to provide more comprehensive and reliable evidence.

The choice of rhEGF as an adjunct to cosmetic debridement and suture in maxillofacial trauma is supported by three key considerations. First, tissue specificity: rhEGF exhibits high affinity for epidermal and mucosal epithelial cells, which are abundant in maxillofacial soft tissues. Unlike PDGF or FGF, which primarily promote fibroblast proliferation and angiogenesis, rhEGF specifically accelerates re‐epithelialization—critical for reducing scar width in exposed facial areas [29]. Second, clinical accessibility: rhEGF is widely available as a topical formulation (e.g., gels, sprays) in both Chinese and international markets, with established regulatory approval for wound care, whereas VEGF or KGF is often limited to experimental settings [30]. Third, cost‐effectiveness: Compared to PRP, which requires in‐house preparation and higher processing costs, rhEGF has lower per‐treatment expenses, making it more feasible for routine use in diverse clinical settings [31].

Notably, included studies consistently used rhEGF (rather than other growth factors), likely due to its documented safety profile in facial applications—minimizing risks of excessive granulation tissue or pigmentation changes, which are concerns with TGF‐β [32].

### 4.1 Optimal rhEGF Administration Parameters

Based on the included studies and existing evidence, key parameters for rhEGF application in maxillofacial trauma are as follows: All six studies initiated rhEGF within 24 h post‐suturing, aligning with preclinical data showing that EGF receptor activation is most effective during the acute inflammatory phase (0–48 h post‐injury) [33]. Topical application (direct gel application to sutured wounds) was universal, avoiding systemic administration to limit off‐target effects (e.g., stimulation of non‐target epithelial cells). Included studies used 50–100 μg/day, with higher doses (100 μg) reserved for wounds > 5 cm^2^, consistent with guidelines suggesting dose titration based on wound area [34]. Treatment duration ranged from 5 to 7 days, reflecting the typical re‐epithelialization timeline for facial wounds; longer courses (≥ 10 days) were not associated with superior outcomes in subgroup analyses [35].

The funnel plot for clinical efficacy showed asymmetry, indicating potential publication bias, possibly due to: Selective reporting (more publication of small positive studies vs. unpublished non‐significant ones); Methodological differences (smaller studies with weaker rigor overestimating effects); Language bias (missed non‐English/Chinese studies with non‐significant findings). To reduce bias, future meta‐analysis updates should include gray literature and trial registries to incorporate unpublished non‐significant studies, enhancing comprehensiveness.

This study has several limitations. First, publication bias was suggested by funnel plot asymmetry, potentially due to selective publication of positive results, as discussed above. Second, our literature search was restricted to English and Chinese databases, which may introduce language bias. Studies published in other languages (e.g., Spanish, German, Japanese) that investigate the same intervention could have been excluded, limiting the generalizability of our findings to populations where these languages are dominant. Third, the number of included studies is small (*n* = 6), and most are single‐center, which may reduce the robustness of the conclusions. Future studies should address these limitations by: (1) Expanding the search to gray literature and trial registries to reduce publication bias; (2) Including databases in multiple languages (e.g., EMBASE for non‐English European studies, Ichushi‐Web for Japanese literature) to minimize language‐related selection bias; (3) Conducting large‐scale, multi‐center randomized controlled trials to validate the observed effects.

## 5 Conclusion

Cosmetic debridement and suture combined with rhEGF has a definite clinical effect in the treatment of maxillofacial trauma, which is helpful in improving the prognosis of patients. Due to the limitations of the included studies, the next research still needs to carry out high‐quality, large‐sample clinical randomized controlled studies to provide more reliable evidence‐based evidence.

## Ethics Statement

The authors have nothing to report.

## Conflicts of Interest

The authors declare no conflicts of interest.

## Data Availability

The data that support the findings of this study are available on request from the corresponding author. The data are not publicly available due to privacy or ethical restrictions.
